# Evaluating Variations of Bladder Volume Using an Ultrasound Scanner in Rectal Cancer Patients during Chemoradiation: Is Protocol-Based Full Bladder Maintenance Using a Bladder Scanner Useful to Maintain the Bladder Volume?

**DOI:** 10.1371/journal.pone.0128791

**Published:** 2015-06-03

**Authors:** Hong In Yoon, Yoonsun Chung, Jee Suk Chang, Joo Yong Lee, Soo Jung Park, Woong Sub Koom

**Affiliations:** 1 Department of Radiation Oncology, Yonsei University College of Medicine, Seoul, Korea; 2 Department of Radiation Oncology, Samsung medical center, Seoul, Korea; 3 Department of Urology, Yonsei University College of Medicine, Seoul, Korea; 4 Department of Internal Medicine and Institute of Gastroenterology, Yonsei University College of Medicine, Seoul, Korea; 5 Yonsei Song-Dang Institute for Cancer Research, Yonsei University College of Medicine, Seoul, Korea; Eberhard Karls University, GERMANY

## Abstract

**Purpose:**

The maintenance of full bladder is important to reduce radiation-induced toxicities and maintain the therapeutic consistency in locally advanced rectal cancer patients who underwent radiotherapy (RT). So, the aim of this study was to evaluate the effectiveness of protocol-based full bladder maintenance by assessing bladder volume variation using an ultrasound bladder scanner to maintain bladder volume.

**Materials and Methods:**

From March 2011 to May 2011, twenty consecutive rectal cancer patients receiving external beam RT participated in this prospective study. Protocol-based full bladder maintenance consisted of education, training and continuous biofeedback by measuring bladder volume. Bladder volume was measured by bladder scan immediately before simulation CT scan and before each treatment three times weekly during the RT period. The relative bladder volume change was calculated. Intra-patient bladder volume variations were quantified using interquartile range (IQR) of relative bladder volume change in each patient. We compared intra-patient bladder volume variations obtained (n=20) with data from our previous study patients (n=20) performing self-controlled maintenance without protocol.

**Results:**

Bladder volumes measured by bladder scan highly correlated with those on simulation CT scan (R=0.87, p<0.001). Patients from this study showed lower median IQR of relative bladder volume change compared to patients of self-controlled maintenance from our previous study, although it was not statistically significant (median 32.56% vs. 42.19%, *p*=0.058). Upon logistic regression, the IQR of relative bladder volume change was significantly related to protocol-based maintenance [relative risk 1.045, 95% confidence intervals (CI) 1.004-1.087, *p*=0.033]. Protocol-based maintenance included significantly more patients with an IQR of relative bladder volume change less than 37% than self-controlled maintenance (*p*=0.025).

**Conclusion:**

Our findings show that bladder volume could be maintained more consistently during RT by protocol-based management using a bladder scan.

## Introduction

Preoperative chemoradiation (CRT) is a standard treatment for locally advanced rectal cancer, which increases local control and sphincter preservation rates compared with adjuvant treatments [1–4]. Additionally, for rectal cancer patients with adverse pathologic factors such as T3-4 or positive lymph nodes, postoperative CRT is required to improve treatment outcome [5]. Generally, pelvic irradiation for rectal cancer patients is safe and tolerable.

Small bowel damage is a common treatment-related complication for rectal cancer patients receiving CRT. It is known that the risk of gastrointestinal (GI) toxicities is related with irradiated small bowel volume and the dose within the radiation field [6]. In some cases, patients develop acute toxicities such as nausea, vomiting, abdominal pain, and abnormal absorption, as well as chronic toxicities including bowel obstruction, perforation, and intestinal stricture. In preoperative CRT for rectal cancer, grade 3 or 4 acute toxicity and diarrhea is reported to develop in 12~36% of patients, whereas long-term GI toxicity occurs in 9% of patients [2,7,8]. Similarly, 18~35% of patients receiving postoperative CRT are reported to have severe acute toxicity and 5~15% of patients have late toxicity related to small bowel damage [2,7,9,10]. Therefore, many investigators have attempted to reduce the irradiated dose of small bowel using non-surgical methods such as the prone position, as well as small bowel displacement devices like the belly board and bladder distension [11,12]. Since 2009, our institution has treated all rectal cancer patients by distending the bladder fully with a belly board in the prone position preoperatively or postoperatively.

However, after CT simulation with a full bladder volume, patients find it very difficult to maintain the bladder volume similar to that of the simulation CT during the entire duration (5 to 6 weeks) of radiotherapy (RT). Some studies using ultrasound bladder scanner for cervical or prostate cancer reported large variations of bladder volume but noted the bladder scanner was useful and accurate to assess the inter-fractional variation of bladder volume [13–16]. Our institution has assessed bladder volume every other day using a bladder scanner since February 2011 and in our previous pilot study, we reported that there were bladder volume variations and reductions in bladder volume in rectal cancer patients receiving CRT [17]. It was previously reported that biofeedback could improve the consistency of bladder volume despite a lack of statistical significance [16]. Thus, the purpose of this prospective study was to evaluate the usefulness of protocol-based full bladder maintenance by assessing variations of bladder volume using an ultrasound bladder scanner to maintain the bladder volume during radiation for locally advanced rectal cancer.

## Materials and Methods

### Patients

This prospective observational study received approval from the Severance internal review boards of the Severance hospital (IRB No. 4-2010-0832). From March 2011 to May 2011, twenty consecutive rectal cancer patients receiving external beam RT participated in this study and provided written informed consent. The inclusion criteria of this study were as follows: age ≥20 years with planned preoperative or postoperative RT for locally advanced rectal cancer. We excluded patients who had urinary frequency of more than once per hour, nocturia more than 4 times per day, urinary incontinence, median laparotomy scar or lymphocele interfering with bladder scanning, aberrant bladder form, or poor compliance. Patient characteristics are listed in Table 1.

**Table 1:** Patient characteristics.

Characteristics		n	(%)
Age (years)	Median	56	
	Range	(22–73)	
Gender	Female	6	(30.0)
	Male	14	(70.0)
Performance status	ECOG PS 0	8	(40.0)
	ECOG PS 1	12	(60.0)
Distance from AV (cm)	Median	7	
	Range	(3–12)	
Tumor location	Lower rectum	8	(40.0)
	Mid rectum	6	(30.0)
	Upper rectum	6	(30.0)
Pathology	Adenocarcinoma	20	(100.0)
Tumor grade	WD	4	(20.0)
	MD	15	(75.0)
	PD	0	(0.0)
	Unknown	1	(5.0)
Aim of radiotherapy	Preoperative	13	(65.0)
	Postoperative	7	(35.0)
Clinical T stage for preoperative RT	T2	2	(15.4)
	T3	10	(76.9)
	T4	1	(7.7)
Clinical N stage for preoperative RT	N0	3	(23.1)
	N1	1	(7.7)
	N2	9	(69.2)
Pathologic T stage for postoperative RT	T2	2	(28.6)
	T3	5	(71.4)
	T4	0	(0.0)
Pathologic N stage for postoperative RT	N0	2	(28.6)
	N1	3	(42.9)
	N2	2	(28.6)
RT dose (Gy)	Median	50.4	
	Range	(45–54)	
Fractional dose (Gy)	Median	1.8	
	Range	(1.8–2.0)	

Patient characteristics are listed.

Abbreviations: ECOG PS = Eastern Cooperative Oncology Group Performance Status Scale; WD = well differentiated; MD = moderately differentiated; PD = poorly differentiated; RT = radiotherapy

### Chemoradiotherapy

CT simulation and treatment were performed with the patient with a full bladder in a prone position with a belly board [18]. All patients received CRT consisting of 3-dimensional conformal RT (3D-CRT). Treatment planning was accomplished with Pinnacle^3^ (Philips Medical Systems, Andover, MA). Target volumes and critical adjacent organs, including the bladder and small bowel, were delineated by the attending radiation oncologists. The clinical target volume included the primary tumor mass or postoperative tumor bed, mesorectum, presacral space, as well as the pelvic lymph nodes and/or external iliac lymph nodes if indicated. For the whole pelvic field, the superior border was defined as the L5-S1 interspace, the inferior border as 3–4 cm below the primary tumor or inferior border of obturator foramen, and the lateral border as 1.5 cm behind the true bony pelvis. For the lateral fields, we defined the anterior border as the posterior margin of the symphysis pubis and the posterior border as the anterior bony sacral margin. In case of a tumor extending directly to a pelvic organ with external drainage, we modified the anterior border to the anterior border of the symphysis pubis to include the external iliac lymphatics. The 3D-CRT consisted of 41.4–45 Gy of whole pelvic RT and 4.5–9.0 Gy of local boost RT to the postoperative tumor bed or primary tumor. The median total dose was 50.4 Gy (range, 45–54). Concurrent intravenous chemotherapy was administered with a 5-fluorouracil (425 mg/m2) and leucovorin (20 mg/m2) bolus on weeks 1 and 5 of 3D-CRT.

### Protocol-based full bladder maintenance by education, training, and continuous biofeedback

Protocol-based full bladder maintenance consisted of education, training and continuous biofeedback by measuring bladder volume. The specific bladder-filling instructions regarding comfortably full bladder were given as follows. First, immediately after patients void the bladder, they drink 500 ml water to have a comfortably full bladder. Second, patients wait until they have a definite but easily tolerable micturition urge (grade 3) or intolerable micturition urge (grade 4). Third, patients record each duration of grade 3 or grade 4 micturition urge in a micturition diary. Fourth, patients visit the hospital at an intermediate status (between grade 3 and 4 of micturition urge) to have a comfortably but maximally full bladder. Until the date of CT simulation, patients were asked to train at least twice according to these specific instructions. Next, all patients were additionally trained three times a week (Monday, Wednesday, and Friday) during the 5- or 6-week RT course according to the biofeedback protocol. The aim of the biofeedback protocol was to improve the constancy of bladder volume measured on bladder scan. We attempted to improve the ability of patients to detect bladder-filling sensations, similar to bladder fullness on simulation CT scan. The feedback consisted of advising patients to drink more water or suppress urination longer according to their daily bladder volume. When the bladder volume of patients ranged from 80% to 120% of that of the simulation CT scan, patients were instructed to keep the same pattern of bladder filling the following day. When the bladder volume was less than 80% of that of the simulation CT scan, patients were instructed to drink 100 or 200 ml more fluid or to suppress urination longer. When the bladder volume was less than 50% of the simulation CT scan volume, patient was advised to suppress urination for 30 minutes to 1 hour. When the measurement was greater than 120% of the simulation CT scan, patients were advised to drink less.

### Bladder volume measurements

Using a portable automated ultrasonic bladder scanner (Biocon-700, Mcube Technology, Korea), bladder volume was measured by two experienced physicians immediately before the simulation CT scan as well as prior to treatment during 5 or 6 weeks of RT. Patients were comfortably positioned in a supine position. Next, the operator placed the scanner probe on two fingers over the symphysis pubis at the midline of abdomen and angled the probe towards the bladder. Operators checked the three-dimensional real time image before scanning to locate the bladder position for more accurate measurement. Each bladder scan time and bladder volume (ml) measured by the bladder scanner was documented. Before simulation CT, the bladder was scanned consecutively 5 times, and the median value was recorded as the baseline bladder volume. The bladder volume was also calculated using the contour of bladder inner wall based on the simulation CT images. During the entire treatment period, each patient’s bladder was scanned 3 days per week (Monday, Wednesday, and Friday) just prior to RT, and 5 times daily to report the median value. Each ultrasound scan required approximately 1–2 min and the addition of an ultrasound scan did not affect overall treatment time.

### Toxicity evaluation and follow-up

Patients were prospectively followed up at 1 and 3 months following the completion of RT, then every 3 months during the first 2 years after RT, and every 6 months from the third year. Treatment-related adverse events were evaluated according to the National Cancer Institute Common Toxicity Criteria (version 3.0).

### Statistical analyses

When bladder volume measurement data was missing for any reason, we replaced the missing values for bladder volume measured using the last observation carried forward (LOCF) method. Eighteen patients (90%) completed planned measurement of bladder volume according to schedule. In two patients, bladder volume measurement data was missing, (one data in one patient and five data in the other). In order to assess the accuracy of bladder ultrasound scan, the correlation between the bladder volume measured by bladder scanner and the bladder volume calculated using simulation CT images was analyzed using Pearson’s correlation tests. The bladder volumes during the RT and the baseline bladder volumes measured by bladder ultrasound scan before CT simulation were compared using Wilcoxon signed-rank tests. To evaluate the protocol-based full bladder maintenance, we compared 20 patients from this study with 20 patients performing self-controlled maintenance without protocol-based education, training and continuous biofeedback from our previous study [17]. The relative bladder volume change of all patients was calculated using the following equation:

$$Relative\;bladder\;volume\;change\;(\%)\;=\;\frac{\text{Bladder volume during RT }\left(\text{ml}\right)-\text{Baseline bladder volume }\left(\text{ml}\right)}{\text{Baseline bladder volume }\left(\text{ml}\right)}\times \text{100}$$

We calculated the interquartile range (IQR) of relative bladder volume change for each patient in this study and each patient in the previous study. Intra-patient bladder volume variations were quantified by IQR of relative bladder volume change in each patient. Next, we compared relative bladder volume change and IQR of relative bladder volume change of each patient between two studies using Mann-Whitney U tests. For further evaluation of the effect of protocol-based full bladder maintenance on improvement of the intra-patient bladder volume variations, we analyzed the correlation between IQR of relative bladder volume change and protocol-based full bladder maintenance using binary logistic regression for a total of 40 patients from this and previous studies. We determined the cut-off value of IQR of relative bladder volume change for protocol-based full bladder maintenance using receiver operating characteristic (ROC) curves. The 40 patients were divided into two groups according to the cut-off value. We performed Chi-square tests to investigate the impact of protocol-based full bladder maintenance on the two groups according to the cut-off value. Statistical significance was defined as *p*<0.05, and SPSS 20.0.0 (IBM Corporation, Armonk, New York, USA) was used for all analyses.

## Results

### Treatment-related acute and chronic toxicity

Most acute and chronic toxicities in this prospective study were mild (grade 0–2). Grade 4 or 5 acute toxicity was not observed in any patient. Eleven of the patients (55%) experienced grade 3 diarrhea, five patients (25%) had acutely grade 3 tenesmus, and one patient (5%) had a grade 3 skin rash (Table 2). Grade 4 or 5 chronic toxicity was also not observed in any patient. No patients experienced grade 3 chronic toxicity, excepting 3 patients (15%) with grade 3 diarrhea (Table 3).

**Table 2:** Prospective evaluation of acute toxicity (n = 20).

	Grade 0		Grade 1		Grade 2		Grade 3		Grade 4	
Nausea	15	75%	4	20%	1	5%	0	0%	0	0%
Vomiting	0	0%	0	0%	0	0%	0	0%	0	0%
Fatigue	3	15%	13	65%	4	20%	0	0%	0	0%
Weight loss	18	90%	2	10%	0	0%	0	0%	0	0%
Anorexia	6	30%	12	60%	2	10%	0	0%	0	0%
Diarrhea	3	15%	1	5%	5	25%	11	55%	0	0%
Tenesmus	9	45%	3	15%	3	15%	5	25%	0	0%
Incontinence, anal	17	85%	3	15%	0	0%	0	0%	0	0%
Skin rash	15	75%	4	20%	0	0%	1	5%	0	0%
Cystitis	17	85%	3	15%	0	0%	0	0%	0	0%
Incontinence, urinary	0	0%	0	0%	0	0%	0	0%	0	0%
Abdominal pain	16	80%	3	15%	1	5%	0	0%	0	0%

The data of acute toxicity are listed.

**Table 3:** Prospective evaluation of late toxicity (n = 20).

	Grade 0		Grade 1		Grade 2		Grade 3		Grade 4	
Nausea	20	100%	0	0%	0	0%	0	0%	0	0%
Vomiting	0	0%	0	0%	0	0%	0	0%	0	0%
Fatigue	14	70%	6	30%	0	0%	0	0%	0	0%
Weight loss	20	100%	0	0%	0	0%	0	0%	0	0%
Anorexia	15	75%	5	25%	0	0%	0	0%	0	0%
Diarrhea	10	50%	3	15%	4	20%	3	15%	0	0%
Tenesmus	15	75%	2	10%	3	15%	0	0%	0	0%
Incontinence, anal	16	80%	4	20%	0	0%	0	0%	0	0%
Skin rash	20	100%	0	0%	0	0%	0	0%	0	0%
Cystitis	20	100%	0	0%	0	0%	0	0%	0	0%
Incontinence, urinary	0	0%	0	0%	0	0%	0	0%	0	0%
Abdominal pain	20	100%	0	0%	0	0%	0	0%	0	0%

The data of chronic toxicity are listed.

### Bladder volume measurement validation

**Fig 1:**
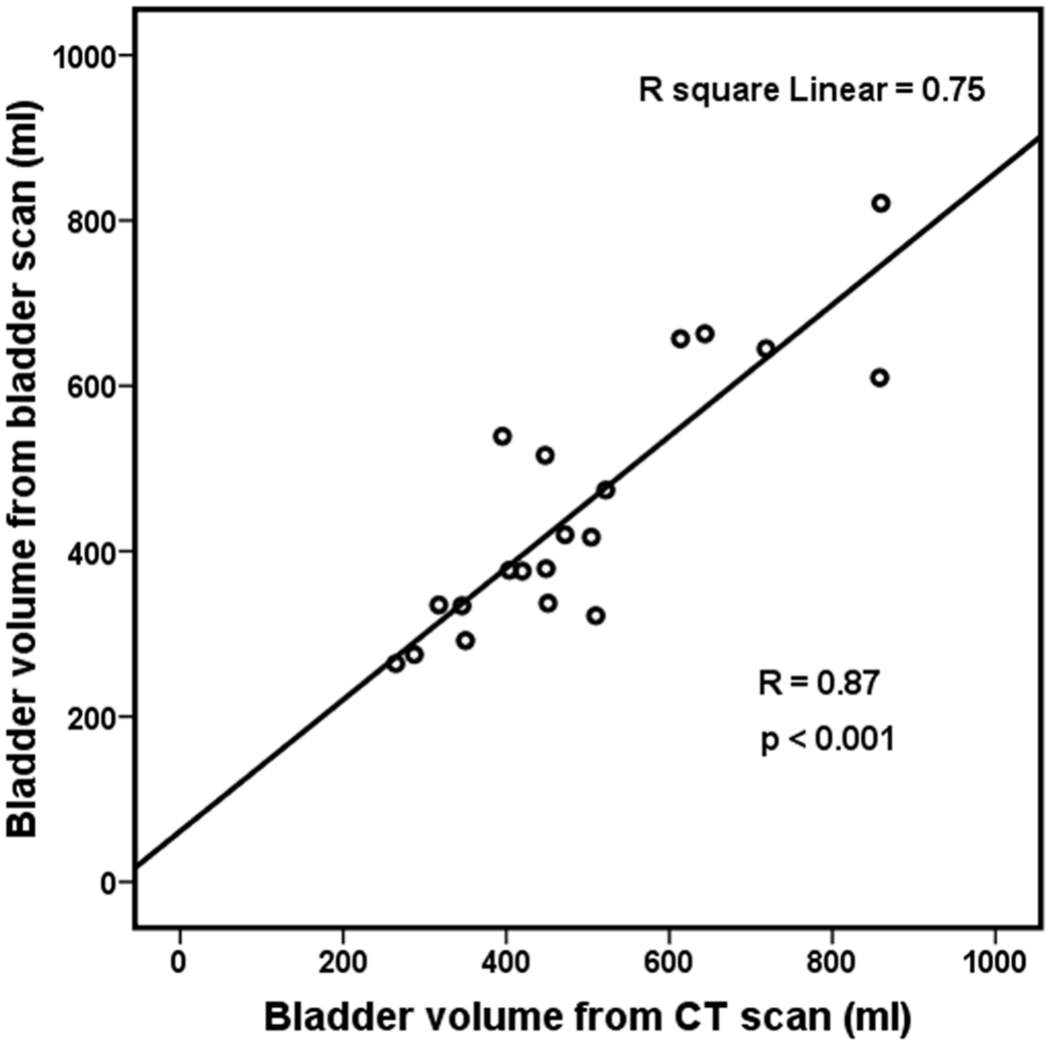
Correlation of bladder volume measured using a bladder scan and simulation CT scan. This figure depicts that bladder volumes measured by bladder scan highly correlated with those on simulation CT scan (R = 0.87, p<0.001).

All patients received bladder ultrasound scans a median of 16 times (range: 15–18; 3 days a week according to the RT schedule). All patients received prearranged scans a median of 16 times (range: 11–18). The median bladder volume on simulation CT image was 450 ml (IQR 195, range: 264–860). The median volume measured by bladder ultrasound scanner before simulation CT scan on the same day was 398 ml (IQR 240; range: 264–821). We observed that bladder volumes measured by bladder scan highly correlated with those on simulation CT scan (R = 0.87, *p*<0.001, Fig 1).

### Time trends of bladder volume measured using bladder ultrasound scan

The bladder volume measurements and the number of patients adjusted by LOCF according to time sequence are described in Table 4. Due to missing data, we used LOCF from V_4-2_ to V_5-3_. Compared to baseline bladder volume by bladder scan, all median values of bladder volumes measured during RT were significantly lower (*p* <0.05), except the first scan after the start of treatment (*p* = 0.087, Table 4).

**Table 4:** Time trends of bladder volume from CT scans and bladder scans.

	No. of patients	Median volume (ml)	IQR	Range (ml)	*p* value^a^
V_CT_	20	450	195	264–860	-
V_B-SCAN_	20	398	240	264–821	-
V_1-1 week_	20	319	234	147–929	0.087
V_1-2 week_	20	306	146	60–734	0.001
V_1-3 week_	20	270	135	50–561	0.001
V_2-1 week_	20	371	161	136–642	0.017
V_2-2 week_	20	300	154	219–536	0.002
V_2-3 week_	20	358	196	173–488	0.01
V_3-1 week_	20	348	166	124–634	0.03
V_3-2 week_	20	268	62	147–606	<0.001
V_3-3 week_	20	317	179	126–557	0.014
V_4-1 week_	20	333	144	117–528	0.006
V_4-2 week_	20	252	182	134–532	0.001
V_4-3 week_	20	272	122	84–482	0.001
V_5-1 week_	20	336	140	147–485	0.003
V_5-2 week_	20	277	182	98–528	<0.001
V_5-3 week_	20	282	130	137–511	<0.001
V_6-1 week_	17	284	65	130–436	0.002
V_6-2 week_	8	335	130	216–560	0.025
V_6-3 week_	7	258	266	149–448	0.018

The bladder volume measurements and the number of patients adjusted by LOCF according to time sequence are described.

^a^ *p* value between VB-SCAN and V_x-x week_

Abbreviations: IQR = interquartile range; VCT = bladder volume measured from simulation CT scans; VB-SCAN = bladder volume scanned by bladder scan prior to simulation CT scans; V_x-x week_ = bladder volume scanned by bladder scan at post-RT_x-x week_.

### Assessment of intra-patient variation according to protocol-based maintenance

**Fig 2:**
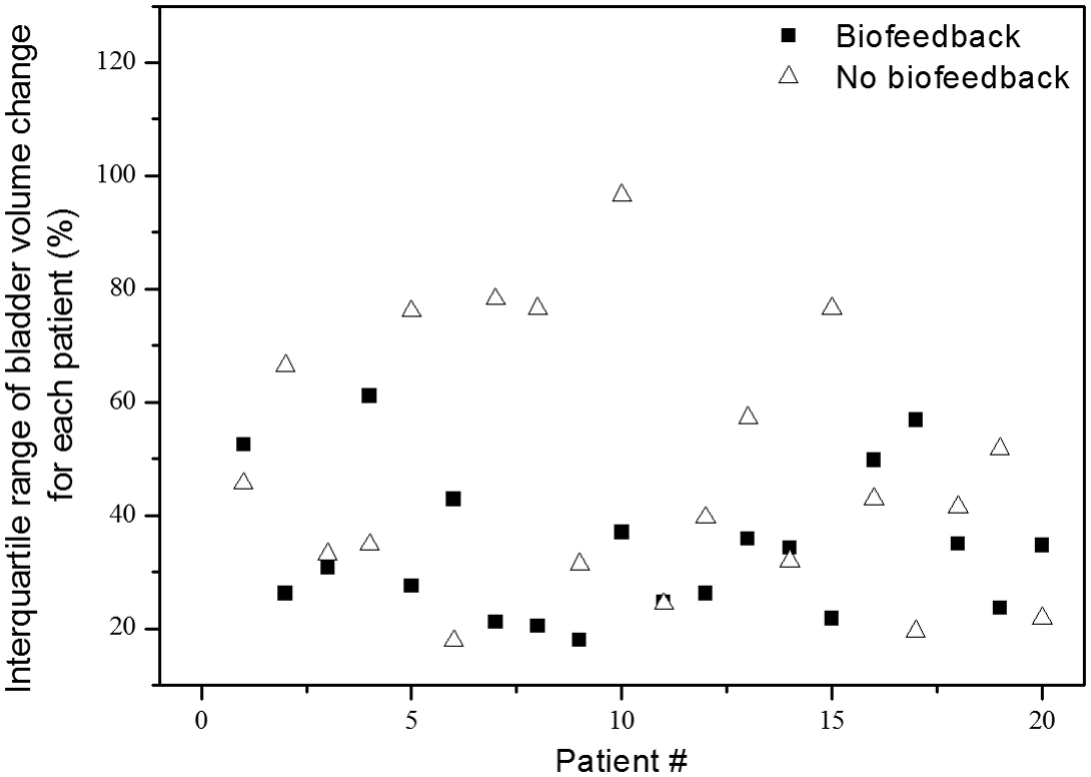
The distribution of interquartile range of relative bladder volume change according to protocol-based maintenance. The interquartile range of relative bladder volume change for patients receiving protocol-based full bladder maintenance (black squares) had smaller variation than that of patients performing self-controlled maintenance (open triangles).

The median values and IQRs of relative bladder volume change of each patient from this and our previous study patients were calculated (Table 5). Comparing median intra-patient relative bladder volume change between current and previous study, protocol-based full bladder maintenance group showed less bladder volume reduction than self-controlled maintenance group although a difference is not statistically significant (median 27.4% reduction in current study vs. 32.9% reduction in previous study, *p* = 0.55). To evaluate intra-patient variation, we investigated the difference between median values of each patient’s IQR of relative bladder volume change between patients receiving protocol-based full bladder maintenance (n = 20) and patients from the previous study who performed self-controlled full bladder maintenance (n = 20). Patients from this study receiving protocol-based maintenance showed lower median IQR of relative bladder volume change although a difference is not statistically significant [median 32.56% (range 18.03–61.09) vs. 42.19% (range 17.79–96.6), *p* = 0.058]. In Fig 2, we observed that patients receiving no protocol-based maintenance had wider IQR ranges of relative bladder volume change. Therefore, to further evaluate the correlation between IQR of relative bladder volume change and protocol-based maintenance, we performed binary logistic regression. Upon logistic regression, the IQR of relative bladder volume change was significantly related to protocol-based maintenance [relative risk 1.045, 95% confidence intervals (CI) 1.004–1.087, *p* = 0.033]. We calculated cut-off values of IQR of relative bladder volume change for protocol-based maintenance using ROC curve analysis (AUC 0.68, 95% CI 0.51–0.85) and determined 37% as a cut-off value (Table 6). Using Chi-square tests, the current study included significantly more patients with an IQR of relative bladder volume change less than 37% than our previous study (*p* = 0.025, Table 6).

**Table 5:** Relative bladder volume change for each patient.

Patient	Current study (protocol-based maintenance)		Previous study (self-controlled maintenance)	
	Median (%)	IQR (%)	Median (%)	IQR (%)
Case 1	-19.66	52.52	-35.10	17.79
Case 2	-40.72	26.17	-56.15	19.49
Case 3	1.71	30.82	-67.60	21.81
Case 4	4.55	61.09	-66.95	24.36
Case 5	5.30	27.46	-68.24	31.42
Case 6	-27.45	42.84	-55.50	31.92
Case 7	-53.96	21.16	-41.99	33.12
Case 8	-31.15	20.49	-9.17	34.86
Case 9	-27.42	18.02	-26.67	39.63
Case 10	-38.17	36.98	-65.69	41.42
Case 11	-18.35	24.73	-26.90	42.95
Case 12	-51.83	26.19	-76.04	45.64
Case 13	-24.04	35.91	23.03	51.69
Case 14	-41.90	34.29	-33.12	57.25
Case 15	-51.55	21.78	-32.43	66.41
Case 16	-35.75	49.74	0.00	76.08
Case 17	7.76	56.83	41.27	76.51
Case 18	-22.91	34.97	-0.88	76.55
Case 19	-15.52	23.58	73.62	78.21
Case 20	-46.20	34.70	77.55	96.60

The median values and interquartile ranges of relative bladder volume change of each patient from this and our previous study patients are described.

Abbreviations: IQR = interquartile range

**Table 6:** Biofeedback effect relative to bladder volume consistency.

Variables	Current study (protocol-based maintenance)	Previous study (self-controlled maintenance)	*p* value
IQR of RBVC <37	15 (75.0)	8 (40.0)	0.025
IQR of RBVC ≥37	5 (25.0)	12 (60.0)	

The current study included significantly more patients with an interquartile range of relative bladder volume change less than 37% than our previous study.

Abbreviations: IQR = interquartile range; RBVC = Relative bladder volume change

## Discussion

The findings from this prospective study showed that although absolute values of bladder volume measured by bladder scans differed from bladder volume on simulation CT scan, there was significant positive correlation between bladder volumes of simulation CT and bladder ultrasound scans. Bladder scan is a useful and accurate device to measure bladder volume easily. During the entire RT course for rectal cancer, there was significant reduction and a wide range of variation in bladder volume compared to baseline bladder volume, despite protocol-based education, bladder filling training and continuous biofeedback. However, this study demonstrated that compared to our previous study, protocol-based full bladder maintenance could reduce the intra-patient variation of bladder volume during the RT course and improve the consistency of bladder volume.

This study has some special considerations. First, this study is an observation study. In addition, we used patients in the preliminary study as a control group, which may lower the quality of data in the control group due to the lack of experience using the bladder scanner in the initial stages of the preliminary study. However, bladder scans and CT bladder volumes were highly correlated in the control group, and patients in the control group fit the inclusion criteria of this study. Therefore, we consider our results are sufficient to show the importance of protocol-based full bladder maintenance. For a more accurate study, further randomized trials are necessary to study the effect of protocol-based maintenance. Second, basic bladder function tests were not performed on patients enrolled in this study. However, we excluded patients with micturition problems and no patient showed micturition problems during the study period. Any patient with mild or severe urinary frequency or nocturia was not included in this study. Thus, the absence of basic bladder function tests in this study likely did not significantly impact the results of this study. Third, there was no significant difference of median relative bladder volume change of each patient between two groups due to small number of enrolled patients. But, we consider that despite small number of patients, our findings showed less bladder volume reduction compared to initial planning and day-to-day variation through protocol-based bladder volume maintenance. Forth, in the era of intensity-modulated RT (IMRT), the protocol-based full bladder maintenance might be less applicable and important since IMRT could reduce radiation-induced toxicity for rectal cancer patients. Nevertheless, we consider that the addition of full bladder maintenance to IMRT could help minimize irradiated dose to organs at risk and radiation-induced toxicity in IMRT for rectal cancer. In this context, further study about the applicability of bladder scanner in IMRT for rectal cancer would be needed. Considering these limitations, properly designed randomized trial with more patients should be needed.

In a previous study from another institute, the usefulness of a bladder scan to achieve better reproducibility of bladder filling during pelvic RT was evaluated [16]. These authors showed that daily bladder volume variation was not significantly improved by biofeedback protocols by comparing the control and feedback group (47.2% and 40.1%, respectively, *p* = 0.2). However, although not statistically significant, bladder volume during RT was better, and daily bladder volume variations were smaller, in the feedback group. This was perhaps due to reasons such as baseline micturition problems of prostate cancer patients. Similarly, another study of prostate patients also reported large bladder volume variance [19]. However, although a small number of patients were evaluated, we enrolled rectal cancer patients without baseline micturition problems according to strict inclusion and exclusion criteria. In addition, we used a more definitive and objective biofeedback protocol. Thus, in rectal cancer patients without baseline micturition problems, we consider that definitive protocol-based continuous biofeedback training of bladder filling using bladder ultrasound scan can improve bladder volume consistency during the RT course, thereby maintaining effective and safe treatment.

Our findings showed that bladder scans were fairly accurate in measuring bladder volume compared to bladder volume on simulation CT scan. Other previous studies have also reported that there was a strong correlation between bladder ultrasound scan and simulation CT scan [14–16]. Because CT scan cannot be performed every day or weekly during the entire treatment period, bladder scans can be utilized as an easy, convenient, and useful tool to check bladder volume and allow steady biofeedback training progress. However, we managed the full bladder maintenance protocol using bladder scans and improved bladder volume consistency during RT, and bladder volume during RT period was still significantly lower than baseline bladder volume. Ahmed et al. also reported that bladder volumes were dramatically reduced during treatment despite of full bladder training [14]. Thus, it is necessary to develop and evaluate additional methods or materials to reduce the differences in absolute bladder volume between baseline and the RT period.

## Conclusions

In conclusion, we report that it would be possible to maintain bladder volume steadily during the entire RT course through protocol-based education, training, and continuous biofeedback and bladder scan utilization. Further study will be required to critically evaluate the influence of protocol-based full bladder maintenance, and additional methods need to be studied for improved consistency of bladder volume.
